# Intuitive Eating Behaviour among Young Malay Adults in Malaysian Higher Learning Institutions

**DOI:** 10.3390/nu15040869

**Published:** 2023-02-08

**Authors:** Rosmaliza Muhammad, Wan Nur Diana Rajab aka Wan Ismail, Syauqina Firdus, Syahrul Bariah Abdul Hamid, Ummi Mohlisi Mohd Asmawi, Norazmir Md Nor

**Affiliations:** 1 Department of Culinary Arts & Gastronomy, Faculty of Hotel and Tourism Management, Universiti Teknologi MARA, Puncak Alam Campus, Puncak Alam 42300, Malaysia; 2 Centre for Dietetics Studies, Faculty of Health Sciences, Universiti Teknologi MARA, Puncak Alam Campus, Puncak Alam 42300, Malaysia; 3 Maternal, Infant & Young Nutrition Research Group, Faculty of Health Sciences, Universiti Teknologi MARA, Puncak Alam Campus, Puncak Alam 42300, Malaysia; 4 Department of Pathology, Faculty of Medicine, Universiti Teknologi MARA, Sungai Buloh Campus, Sungai Buloh 47000, Malaysia; 5 Integrative Pharmacogenomics Institute, Universiti Teknologi MARA, Puncak Alam Campus, Puncak Alam 42300, Malaysia

**Keywords:** young adult, obesity, intuitive eating, weight-control behaviour, eating disorder, binge eating

## Abstract

Despite the significance of dietary knowledge interventions, there is a lack of established studies on intuitive eating behaviour among young Malay adults in Malaysia. This cross-sectional study aimed to determine the intuitive eating score, identify the intuitive eating factors, and determine the association of intuitive eating with weight-control behaviours and binge eating. A total of 367 respondents completed self-administered questionnaires on sociodemographic characteristics, namely the Intuitive Eating Scale (IES-2) and The Diabetes Eating Problems Survey (DEPS). The findings reported IES-2 mean scores of 3.52 ± 0.32 and 3.47 ± 0.35 for both men and women. No difference in total IES-2 scores was found between genders for Unconditional Permission to Eat (UPE) and Reliance on Hunger and Satiety Cue (RHSC) subscales (*p* > 0.05). However, among all four subscales of IES-2, there was a gender difference in the mean EPR and B-FCC subscale scores (*p* < 0.05). A statistically significant difference was found in intuitive eating, which refers to a belief in one’s body’s ability to tell one how much to eat, in women across living areas (*p* < 0.05). The result shows that there is a relationship between weight-control behaviour and binge eating and dieting, with the coefficient of the relationship (R^2^) of 0.34. As a result, intuitive eating throughout young adulthood is likely to be related to a decreased prevalence of obesity, dieting, poor weight-management behaviours, and binge eating.

## 1. Introduction

Obesity is a problem in both developing and industrialised countries, including Malaysia. Malaysia has the highest rate of obesity and overweight in Asia, with 64% of males and 65% of females being obese or overweight [1]. This can be seen from previous reports that the prevalence of obesity in Malaysia has an upward trend [2,3,4,5]. In 1996, the Second National Health and Morbidity Survey (NHMS) stated that the national prevalence of overweight and obesity was 16.6% and 4.4%, respectively, and the mean body mass index (BMI) of Malaysian adults at that time was 22.48 kg/m [3]. In the third NHMS [4], the national prevalence of overweight among the Malaysian adults was 29.1%, and that of obesity was 14.0% [3]. These figures show that in less than 20 years, the proportion of overweight adults has doubled, whereas the obese proportion has tripled in Malaysia.

In the NHMS [2], the prevalence of obesity among females had doubled (29.6%) since a previous report, and this prevalence was also found to be higher that of than men (25.0%) [6]. Findings from both NHMS [2,4] indicated that obesity among women was found to be higher than among men. In addition, ref. [6] again revealed that the mean body mass index (BMI) among housewives was also higher than other job categories (Mean BMI: 26.6 kg/m^2^). Furthermore, in the NHMS 2015 findings, Malaysia was classified as an obese nation, with the population aged between 18 years and above being the major group of people with overweight and obesity problems, with one out of every two adults being overweight or obese. 

Overall, an increase in obesity percentage has been statistically demonstrated, as the prevalence of obesity rose from 10.5 percent in 2010 to 13.3 percent in 2014, with a peak of 18 percent in 2015. Meanwhile, 5.5 million Malaysians, or 30.6 percent of the population, were obese in 2018. Malaysia is the most obese country in Southeast Asia, with 44.2% of its male and female citizens having a body mass index (BMI) of more than 25 kg/m^2^ [7]. Furthermore, what is even more concerning, as UNICEF discovered in 2016, is that Malaysia is a Southeast Asian nation with prevalent nutritional problems such as overweight and malnutrition among its young adult and children [8]. Moreover, ref. [9] reported that at 15.6 percent in 2019, Malaysia had the highest rate of obesity among Asian countries. 

Many scholars in Malaysia have conducted studies on the obese population related to dietary knowledge, awareness, patterns, and practice intervention programs to explore in-depth related issues on obesity. A sizeable amount of other studies have taken place looking into various aspects of this topic, including obesity intervention programmes [10]; the impact of obesity on pulmonary functions among young non-smoker healthy females in Shah Alam, Malaysia [11]; eating patterns and prevalence of obesity from the Malaysian Food Barometer [12]; a cross-sectional analysis of obesity among adolescents and young adults in Malaysia [13]; prevalence and socio-demographic determinants of overweight and obesity among Malaysian adults [14]; cardio-metabolic health risks in indigenous populations of Southeast Asia and the influence of urbanisation [15]; prevalence and determinants of overweight, obesity, and Type 2 Diabetes Mellitus in adults in Malaysia [16]; and metabolic syndrome on the association of obesity and unhealthy lifestyle among Malaysian elderly people [17]. 

Despite the importance of the dietary knowledge intervention, there is a lack of research on intuitive eating behaviour among Malaysian young adults. Given the nature of intuitive eating, intuitive eating behaviour will be associated with a lower BMI. Self-regulation, for instance, provides a basis for performing a targeted behaviour that could hinder emotional eating. To eat in line with bodily needs, there is a constant demand for attention regulation and monitoring. Therefore, it is believed that a high level of self-regulation could support intuitive eating behaviour. In terms of current findings, most of the existing overweight and obesity intervention was targeted at young adults without considering the different psychological states, cultures, religions, and ethnicities of the young adults, making the current approaches less suitable for Malaysian young adults. Therefore, it is important to understand how the obese population, especially among young adults, benefits from the comprehensive, intuitive-eating behaviour approach. 

Therefore, to explore the related issue in depth, an intuitive eating behaviour approach was applied. Thus, this study looked into findings on the intuitive eating behaviour for obese populations to encourage more studies to be conducted in the future because food intake is not the only way to control and prevent overweight and obesity to ensure a good health and social well-being of the nation. 

Intuitive eating (IE) is defined by a strong link between internal physiological hunger and satiety cues. IE is measured primarily based on the Intuitive Eating Scale-2, which is shortened to IES-2 [18]. The IES-2 measures four characteristics of intuitive eating. The term “Unconditional Permission to Eat” (UPE) refers to the practise of eating whatever meals one desires at the time [19]. “Physical Eating Rather Than Emotional Eating” (EPR) evaluates the people’s capability to eat when physically hungry instead of taking food as a coping tool for unfavourable feelings. In addition, the “Reliance on Hunger and Satiety Cues”, referred to as RHSC, is the competence to regulate food intake based on hunger and satiety cues. The perception of physiological conditions is RHSC’s defining characteristic. “Body-Food Choice Congruence” or B-FCC, on the other hand, refers to selecting palatable and nutritious food by physical requirements [18]. Ref. [20] revealed that a weight loss intervention uses low-calorie meals in fixed serving sizes and improves the participant’s diet quality. As a result, intuitive eating appears to be a feasible alternative to dieting for weight maintenance.

Intuitive eating is an approach for improving one’s eating habits. Intuitive eating, alone or in combination with dietary requirements, had no effect on the overall dimensions of eating behaviours, weight, and BMI in obese adults [21]. The image conveyed by Instagram of mindful and intuitive eating depicts healthy lifestyles without a focus on weight, yet it lacks demographic and body-type variety. Instagram can help health practitioners convey culturally/demographically diverse, evidence-based health/nutrition information to young people [22]. Recently, it suggested that in female-identifying undergrads, intuitive eating intervention may help reduce disordered eating risk variables by decreasing dietary constraint and boosting intuitive eating [23].

The association between BMI and intuitive eating is still explored. Researchers found that a lower BMI was connected with a higher IE score (BMI). People with a higher BMI may be more likely to neglect their physiological signals in favour of external dietary restrictions or at times of stress [24]. These findings suggest that IE may inhibit weight gain. However, according to ref. [25], there was no relationship discovered between BMI and intuitive eating in young adult women. Hence, more studies need to be conducted to examine this relationship.

The majority of intuitive eating research has focused on adult females. In cross-sectional studies on intuitive eating, intuitive eating scores varied according to gender [26,27,28]. The total IES-2 score and subscales for women were significantly lower (except for the Reliance on Hunger and Satiety Cues score). According to reports, women are more in tune with their bodies in terms of hunger and fullness than men [26]. Moreover, exploring intuitive eating in adult males is crucial due to the focus on appearance, weight control, and increased disordered eating behaviours among males [29,30]. Therefore, more studies need to be conducted to investigate this relationship.

Eating disorders such as binge eating can lead an individual to lose touch with their internal cues for hunger and fullness. Intuitive eating, which emphasises selecting when and how much to eat based on bodily hunger and satiety cues, is one method used to improve healthy eating [18]. In both genders, intuitive eating has been related to a lower prevalence of dieting, unhealthy weight control methods, and binge eating in the literature [31]. Previous research has indicated that intuitive eating is also related or associated with food addiction and binge eating [32]. In addition, those who scored well on intuitive eating also had lower scores on the eating disorder scale. Thus, their chance of getting an eating disorder was lower. Individuals who consume food intuitively have a greater nutrient intake and healthier eating behaviours. However, there is a lack of established studies on intuitive eating in Malaysia, and most studies were conducted in Western countries. Given their diverse socio-cultural backgrounds, it is uncertain if findings from Western populations apply to the Malaysian people. Therefore, this study aims to determine the intuitive eating score, identify the intuitive eating factors, and determine the association of intuitive eating with weight-control behaviours and binge eating among young Malay adults.

## 2. Materials and Methods

### 2.1. Study Design

This research utilised a quantitative method through primary data collection via an online survey platform, Google Forms. This study was cross-sectional in that the data were collected and analysed at a specific time. This research was approved by Universiti Teknologi MARA (UiTM), Malaysia Research Ethics Committee (REC); the reference number was REC/04/2022 (ST/MR/61). Primary data collection was conducted via an online survey platform, Google Form. The authors obtained the contact information through the official UiTM email account. The link to the Google Form was blasted via several online platforms such as WhatsApp, Instagram, and email. This study was conducted online from 20 June 2022 and ended in July 2022 through the distribution of a questionnaire on Google Form targeting university students across Malaysia from both public and private universities. The initial target was 250 young adults. The response rate was a bit slow initially, but after the authors approached potential respondents via email, the response rate was fast and good. The non-responders were followed up by sending a reminder for them to answer the survey. The authors continued collecting a number of the respondents by distributing the link of the survey daily through WhatsApp, Instagram, and email.

### 2.2. Inclusion & Exclusion Criteria

The participants were citizens aged 18 to 24 from higher learning institutions in Malaysia, which comprised both public and private universities. Selected participants had no physical or mental disabilities and high literacy, so they could understand both Malay and English. In contrast, the exclusion criteria in this study were non-Malaysian citizens, Malaysian students studying abroad, and individuals with physical or mental disabilities.

### 2.3. Sample Size

The formula below was used to calculate the targeted sample size:

n = [Z^2^ P(1 − P)]/d^2^

where

n = sample size;
Z = Z statistic for a level of confidence (1.96);
P = expected prevalence or proportion (0.5);
d = precision (0.05);
Z = 1.96, P = 0.5, d = 0.05.

Thus,

n = [1.96^2^ (0.5)(1 − 0.5)]/0.052^2^ = 384.16 @ 385

The confidence level will be 95%, as it is assumed to be conventional; thus, the Z value will be 1.96 (Naing et al., 2006) [33]. The *p*-value will be 0.5, with a precision of 0.05. This study involved 385 participants.

### 2.4. Intuitive Eating

The questionnaire was validated by 12 experts in nutrition and behaviour studies to obtain their opinions on the questions that were included in the questionnaire. Permission was obtained before the validation process started. All the comments and suggestions made were used to improve the validity of the questionnaire. Overall, 24 validated items adapted from [18]’s Intuitive Eating Scale-2 (IES-2) were used to determine intuitive eating by measuring respondents’ ability to trust and eat in response to internal hunger and satiety cues while picking foods that they enjoy and function well with their body [18]. The items were assessed on a 5-point scale ranging from 1 (strongly disagree) to 5 (strongly agree), with higher mean scores for the overall scale indicating more intuitive eating. Unconditional Permission to Eat (UPE), Eating for Physical rather than Emotional Reasons (EPR), Reliance on Hunger and Satiety Cues (RHSC), and Body–Food Choice Congruence were the four sub-scales in IES-2 (B-FCC). Two components from the 23-item validated Intuitive Eating Scale were used to investigate aspects of intuitive eating. Participants were asked to rate their satisfaction with the following two statements on a five-point Likert scale: “I trust my body to tell me how much to eat” and “I stop eating when I am full” (test-retest r = 0.67 [first question], r = 0.53 [second question]). To compare intuitive eaters to all other participants, replies were divided into two categories: no (strongly disagree and disagree) and yes (agree and strongly agree). These variables were used independently throughout the analysis. 

### 2.5. Weight Control Behaviour and Binge Eating

Dieting frequency was measured by asking how many times the participant had been on a diet in the previous 12 months. By “diet”, we imply modification of eating habits in order to lose weight. Response options included: never, 1–4 times, 5–10 times, more than 10 times, and always. Responses were dichotomized for this analysis to compare chronic dieters (5 or more times) with those who diet less regularly or not at all (test-retest r = 0.89) [30]. 

Weight control behaviours were rated as healthy or unhealthy based on health promotion and healthy weight maintenance criteria [31]. Six healthy weight-management behaviours were assessed by asking how frequently they were employed to reduce or avoid weight gain. There were five response options ranging from never to regularly (1–4 times, 5–0 times, more than 10 times, and always), followed by the following behaviours: exercise, eating fruits and vegetables, eating less high-fat foods, eating less sweets, drinking sugar-sweetened beverages (SSB), and controlling portion sizes [34]. Similar to previous research findings [35], those who reported engaging in one or more of the six actions were classified as practising healthy weight-management behaviours. 

To assess whether people engage in unhealthy weight-control behaviours, we evaluated if they performed the listed behaviours to reduce or maintain their weight. Fasting to regulate body weight, consuming relatively little food, using meal-replacement products (powder or a special drink), skipping meals, using diet pills, vomiting, and using laxatives and diuretics were among the queries listed. A binary scale was utilised. Those classified as engaging in unhealthy weight-management behaviours were those who reported engaging in one or more unhealthy weight-management behaviours [34]. Binge eating with a lack of control was measured using a single question modified from the validated adapted The Diabetes Eating Problems Survey (DEPS) (2) [34]: “In the last year, have you ever overeaten, most likely your favourite food?” A dichotomous scale was applied, and those who responded yes to the question were considered binge eaters. The dichotomous variable was used for the questionnaire of The Diabetes Eating Problem Survey (2) to assess weight-control behaviours but was not used for the measurement of intuitive eating.

### 2.6. Body Mass Index

BMI was determined based on self-reported height and weight. A BMI of 18.5 kg/m^2^ is regarded underweight, a BMI of 25 to 29.9 kg/m^2^ is considered overweight, and a BMI above 30 kg/m^2^ is considered obese. 

### 2.7. Statistical Analysis

IBM SPSS Statistics 24.0 was used to analyse all of the data. Descriptive statistics were provided for each variable (mean, standard deviation, frequency, and percentage). In descriptive analyses, the independent samples *t*-test measured the difference in intuitive eating scale scores between both genders. The differences in intuitive eating practices by race, living area, and BMI status were analysed using Chi-square tests of significance. Multiple linear regression analysis was applied to determine the association of intuitive eating with weight-control behaviours and binge eating. The statistical significance level was set at *p* < 0.05.

For the validated and standardised self-report measures for intuitive eating behaviours, each of the variables and scales used that are suitable, with some modification, for use with young adults in the self-administered questionnaire were validated by 11 expert panels in the area of nutrition to improve the reliability and validity of the instruments. All the suggestions and recommendations that focus more on Malay language translation were amended accordingly. 

## 3. Results

### 3.1. Sample Characteristics

This survey included 367 undergraduate students, 59 males (16.1%), and 308 females (83.9%), ranging in age from 18 to 24 years old (mean: 22.17, standard deviation: 1.59). Table 1 summarises the participants’ characteristics by gender, race, marital status, educational level, residing area, kind of habitation, and body mass index, which was determined using self-reported height and weight. *n* = 308. 

### 3.2. Intuitive Eating Scale (IES-2) Score 

Table 2 shows the total mean IES-2 score of the respondents as well as four subscale elements of the Intuitive Eating Scale (IES-2): Unconditional Permission to Eat, Eating for Physical Reasons, Reliance on Hunger and Satiety Cues, and Body–Food Choice Congruence. The total IES-2 scores for males and females were 3.52 and 3.47, respectively. Neither males nor females scored 4 or higher on the IES-2. EPR and B-FCC scores were higher in men than in women, and UPE and RHSC scores were higher in women than in men. There was no gender difference in the mean score of the total IES-2 score, UPE, and RHSC subscales (*p* > 0.05). Among all the four subscales of IES-2, there was a gender difference in the mean EPR and B-FCC subscale scores (*p* < 0.05). The Cronbach’s alpha for intuitive eating is 0.74.

UPE stands for unrestricted permission to eat. EPR stands for eating for physiological as opposed to emotional reasons; reliance on hunger and satiety cues is represented by RHSC; B-FCC stands for body–food choice congruence. IES-2 stands for intuitive eating scale-2.

### 3.3. Aspects of Intuitive Eating

Men said they trusted their bodies to tell them how much to eat 64.4% of the time, and 89.8% stated they stopped eating when they were full (Table 3). Among women, 71.8% said they trusted their bodies to tell them how much to eat, and 85.4% said they stopped eating when they were full (Table 3). There was no statistically significant difference in either aspect of intuitive eating by age or race (*p* > 0.05). Both aspects of intuitive eating showed no significant difference in the body weight status of both men and women (*p* > 0.05). There was no statistically significant difference in two characteristics of intuitive eating in men across living environments (*p* > 0.05). However, there was a significant difference in intuitive eating for women across living locations, as women across living locations trusted their bodies to tell them how much to eat (*p* < 0.05).

Two variables were used to assess intuitive eating: trust body (“I trust my body to tell me how much to eat.”) and stop full (“I stop eating when I’m full.”). The percentages shown represent the percentage of people who agreed or strongly agreed. Each aspect of intuitive eating by sociodemographic variable was analysed by Chi-square tests of significance, where *p* values represent discrepancies in the proportion of each gender, reporting each component of intuitive eating with each subgroup (race, area of residence, and BMI).

### 3.4. Dieting, Weight Control Behaviour, and Binge Eating

In our study, 9.8% of men and 52.3% of women were currently dieting. Fewer men (16.1%) than women (83.9%) were involved in healthy weight-control behaviours. Meanwhile, 76.3% of men and 80.7% of women were in unhealthy weight-control behaviours. More women (84.7%) practised binge eating with a loss of control compared to men (81.4%) (Table 4).

### 3.5. Association of Intuitive Eating with Dieting, Weight Control Behaviours, and Binge Eating

Multiple linear regression showed that all the variables were statistically significant (*p* < 0.05), indicating that higher IES-2 scores were associated with dieting, weight-control behaviours, and binge eating (Table 5). 

The result shows a relationship between weight-control behaviour and binge eating towards diet. The coefficient of the relationship (R^2^) of 0.34 shows that 34.0 percent of the diet is explained by the respondents’ weight-control behaviour and binge eating. Furthermore, the effect size of the study is 0.66. 

## 4. Discussion

Overall, there were gender differences in the intuitive eating score, including for UPE and RHSC. The overall IES-2 score and its subscales were considerably lower for women than men. These results were in line with the findings of another study [24], which showed that men’s scores were higher than women’s on the IES-2 scale. The current study found a sex difference in the mean EPR and B-FCC subscale scores (*p* < 0.05). A study by [18] contradicted these findings. Their results reported that males (M = 3.72, SD = 0.84) did not score significantly higher in the EPR subscale score compared to females (M = 3.18, SD = 0.85, *p* > 0.05). Therefore, compared to females, males demonstrated more of a tendency towards intuitive eating and eating for physical rather than emotional reasons than women.

In one study, the researchers examined adolescent gender differences in intuitive eating; females were more inclined than males to manage their emotions through food [36]. However, this study showed that males scored higher in the EPR subscale score (M = 3.10, SD = 0.45) compared to females (M = 3.01, SD = 0.57, *p* < 0.05), which contradicted the findings of a study conducted by [32]. Early emotional relief can explain what eating provides for young males regarding anxiety, loneliness, and boredom. Young adults who ate primarily for physical reasons reported less acceptance of cultural expectations about looks, less pressure to diet and maintain a slender physique, less negative emotions, more positive emotions, and higher satisfaction with their bodies and life. Adolescents may become self-conscious about their bodies, lives, and themselves when they are criticised about their appearance and encouraged by family and friends to diet and lose weight. They may turn to food in these circumstances to help them deal with these pervasive stressful conditions and ease their bad feelings. 

Women scored lower on Eating for Physical Reasons in another survey of college students at Gumushane University’s Faculty of Health Sciences in Turkey. They also ate less for Body–Food Choice Congruence, meaning women had less desire to eat nutritious food than men [37]. This finding aligns with the findings of the current study in that men scored higher in the B-FCC subscale score (M = 3.77, SD = 0.67, *p* < 0.05) compared to women (M = 51, SD = 0.76). It was statistically demonstrated that men were more prone to consuming healthy foods that would enhance their bodies’ performance while also providing them energy and stamina. However, findings from the current study showed no gender difference in the mean score of EPR and B-FCC subscale scores (*p* > 0.05). This result was similar to previous studies by [28,38], which showed that gender was unrelated to the intuitive eating score.

According to the current study, young adults often trust their bodies to tell them how much to eat and when to stop eating. More women (71.8%) than men (64.4%) said they trusted their bodies to tell them how much to eat. To the best of our knowledge, this is the first study to look at features of intuitive eating across sociodemographic variables in Malaysian young adults. A study in Minneapolis also investigated this topic. In research from [28], young Native American men were less likely to quit eating when they were full (61.3%) than young men of other races such as White, Black, Latino, and Asian American. Approximately 60-80% of participants said they trusted their bodies to tell them how much to eat and stopped eating when they were full.

The occurrence of intuitive eating was significant in this sample of adolescents and young adults from 31 Minneapolis public schools, with 50–80% of overweight and obese participants reporting at least one of the measured features of intuitive eating. The prevalent food environment and lifestyles that could account for this trend in their community were not always conducive to permitting food choices and eating behaviours that meet the body’s physiological cues and demands. If individuals fail to meet their body’s cues due to the environmental structure, they might eat whatever they can without considering whether they are hungry, which could lead to weight gain over time [28].

This study discovered minor changes in intuitive eating behaviours based on sociodemographic factors except age, gender, marital status, education level, and residential type. In that study, approximately 60–90% of the respondents trusted their bodies to tell them how much to eat and stopped eating when they were full. One explanation is that only two of the 23 items on the Intuitive Eating Scale are used to measure intuitive eating. When these two aspects are examined separately instead of iteratively, the prevalence of these specific aspects is substantially higher than when intuitive eating is calculated as a whole.

The researchers found that for women, believing in one’s body’s ability to tell one how much to eat was associated with the area of living (*p* < 0.05). Up to this point, no study has examined the aspects of intuitive eating across the living area. However, one study examined intuitive eating among rural Australian adults [38]. According to the study, over 9% of women and 26% of men in rural Australia reported eating intuitively. Because different metrics have been employed in various studies, it is difficult to say how this outcome relates to levels of intuitive feeding in other populations. In their study, a “intuitive eater” was defined as anyone who received an average score of 4 or higher on the Intuitive Eating Scale, as provided by the measure’s author. Furthermore, in our study, the intuitive eater is defined as an individual with a mean score of 4 and above on the total Intuitive Eating Scale-2 (IES-2) score. 

The current study found that neither feature of intuitive eating was associated with a decreased BMI (*p* > 0.05). Each aspect of intuitive eating was less likely in young adult males and females with higher BMI categories. Furthermore, the current study’s findings were similar with previous research [25], in which researchers discovered an inconsequential association between body weight status and intuitive eating. Researchers also examined associations between intuitive eating and health in a study that included college women [39]. Similarly, in a related study, intuitive eating was found to be positively related to HDL cholesterol and adversely related to BMI, triglycerides, and cardiovascular risk. In contrast to the current study’s findings, a different study revealed a positive association between intuitive eating and lower BMI. However, the current study’s findings contradict previous research, as published in [28]. They discovered that certain components of intuitive eating were linked to lower BMI. This is in line with theories that suggest that people who adhere to restricted diet rules are more likely to participate in weight-gaining behaviours, such as eating when they are not physically hungry or binge eating, than those who are more aware of physiological signs of hunger and fullness and eat in response to these signals. Chronic dieters may not recognise when they are hungry or full because their bodies’ natural cues to eat or stop eating have been suppressed by external diet guidelines [40].

This study aimed to investigate the relationship between intuitive eating and participation in various weight-management behaviours in a large sample of young adults, with a mean age of 22.17 years for men and 22.22 years for women. In addition, the current study investigated the relationship between intuitive eating and weight-control approaches that promote health (e.g., exercising and eating less sweets). There was a weak correlation discovered between intuitive eating and appropriate weight-control behaviour. The result shows a relationship between weight-control behaviour and binge eating towards diet. Eating less sweets and eating more fruits and vegetables, as the questions were asked in the diet section of the questionnaire, showed an awareness of a healthy diet. Individuals may also participate in intuitive eating as an alternative to these behaviours [31].

According to the data, intuitive eating was not significantly connected with unhealthy weight-management behaviours (*p* > 0.05), indicating that intuitive eating did not reduce the prevalence of these problematic behaviours. Gender variations in intuitive eating awareness may also be connected. A 2019 research found that 42% of women and 30% of men are familiar with “mindful or intuitive eating.” [41]. Because women frequently participate in more dieting and restrictive eating behaviours than males [42,43], recognising intuitive eating as an alternative diet or incompatible with dieting will be critical in creating knowledge and developing intuitive solutions. Participation in unhealthy weight-control behaviours was already high among women in this study (80.7%), making identifying a rise in incident behaviours difficult. A further study examining the start of involvement in weight-management behaviours and intuitive eating should be conducted to give insight into intervention timing.

There has been little research into the relationship between intuitive eating and binge eating. In a study by [35], intuitive eating was inversely related to binge eating symptoms. Previous cross-sectional research in the United States found correlations between intuitive eating and decreased binge eating in a community-based population [44] and a countrywide selection [45]. However, these findings were contradicted by the current study, in which the researchers found no statistically significant relationship between intuitive eating and binge eating. Thus, intuitive eating did not predict the decreased incidence and prevalence of binge eating among the Malay men and women studied. Because none of the participants in this study was intuitive eaters, they may be more easily disengaged from bodily hunger cues, leading to uncontrollable behaviours like binge eating. As a result, being an intuitive eater may be a useful coping method that reduces the likelihood of binge eating [46].

## 5. Conclusions

The current study indicated that all respondents from both genders were not labelled as “intuitive eaters”. It was believed that Malay young adults did not practice intuitive eating, as the findings from the full IES-2 scale reported a low score. This lower score in intuitive eating, indicating non-intuitive eaters, was found; thus, intuitive eating did not predict the decreased incidence and prevalence of binge eating and unhealthy control behaviour among the Malay men and women studied. The biggest limitation of this research is the disproportion of male and female gender in relation to the student population; 59 male and 308 female, yielding a total value of 367 Malay undergraduate students. This disproportion of males and females could have made it difficult to detect an association between intuitive eating, weight-control behaviour, and binge eating. Future research is needed to determine whether intuitive eating significantly influences body mass index, weight-related behaviours, and psychological well-being; thus, these new findings could lead to the development of interventions to assist adolescents in maintaining a healthy weight and weight-related behaviours. Eating intuitively may be a healthier, more natural alternative to traditional diets and weight-loss approaches.

## Figures and Tables

**Table 1:** Socio-demographic data and anthropometric indices of respondents (*n* = 367).

Demographic Characteristics	Total *n* (%)	Male, % (*n*) *n* = 59	Female, % (*n*) *n* = 308
Age: mean ± SD (years old)		22.17 ± 1.68	22.22 ± 1.58
Race			
Malay	347 (94.6)	54 (14.7)	293 (79.8)
Others	20 (5.4)	5 (1.4)	15 (4.1)
Marital status			
Single	364 (99.2)	58 (15.8)	306 (83.4)
Married	3 (0.8)	1 (0.3)	2 (0.5)
Level of education			
Foundation	17 (4.6)	5 (1.4)	12 (3.3)
Diploma	82 (22.3)	15 (4.1)	67 (18.3)
Degree	265 (72.2)	39 (10.6)	226 (61.6)
Master’s	3 (0.8)	0 (0.0)	3 (0.8)
Area of living			
Urban	137 (37.3)	19 (5.2)	118 (32.2)
Semiurban	182 (49.6)	34 (9.3)	148 (40.3)
Rural	48 (13.1)	6 (1.6)	42 (11.4)
Residential			
Landed	310 (84.5)	48 (13.1)	262 (71.4)
High-rise building with a lift	36 (9.8)	10 (2.7)	26 (7.1)
High-rise building without lift	21 (5.7)	1 (0.3)	20 (5.4)
Body mass index (BMI) (kg/m^2^)			
Underweight	66 (18.0)	10 (2.7)	56 (15.3)
Normal weight	150 (40.9)	21 (5.7)	129 (35.1)
Overweight	94 (25.6)	18 (4.9)	76 (20.7)
Obese	57 (15.5)	10 (2.7)	47 (12.8)

Results are tabulated in mean and standard deviation.

**Table 2:** Comparison on the intuitive eating measure scores of men and women. (*n* = 367).

Scale Score	Men (*n* = 59)	Women (*n* = 308)	t-Value	*p*-Value
UPE subscale score	3.31 ± 0.50	3.33 ± 0.56	0.886	0.376
EPR subscale score	3.10 ± 0.45	3.01 ± 0.57	1.084	0.040
RHSC subscale score	3.63 ± 0.52	3.68 ± 0.53	−0.701	0.484
B-FCC subscale score	3.77 ± 0.67	3.51 ± 0.76	2.492	0.013
Total IES-2 score	3.52 ± 0.32	3.47 ± 0.35	1.454	0.147

The independent samples *t*-test measured the difference in intuitive eating scale scores between both genders. *p* value at <0.05.

**Table 3:** Intuitive eating by sociodemographic characteristics and weight status (*n* = 367).

	Men (*n* = 59)			Women (*n* = 308)		
	*n*	Trust Body (%)	Stop Full (%)	*n*	Trust Body (%)	Stop Full (%)
Overall	59	64.4	89.8	308	71.8	85.4
*Race*						
Malay	347	59.3	81.4	293	68.5	80.8
Others	20	5.1	8.5	15	3.2	4.5
		*p* = 1.00	*p* = 1.00		*p* = 0.77	*p* = 0.71
*Area of living*						
Semiurban	34	37.3	50.8	148	31.5	40.6
Urban	19	18.6	28.8	118	30.5	32.8
Rural	6	8.5	10.2	42	9.7	12.0
		*p* = 0.62	*p* = 1.00		*p* = 0.04	*p* = 0.84
*Body mass index, BMI*						
Underweight	10	11.9	15.3	56	14.3	15.9
Normal weight	21	23.7	30.5	129	29.2	36.7
Overweight	18	20.3	28.8	76	19.2	20.8
Obese	10	8.5	15.3	47	9.1	12.0
		*p* = 0.83	*p* = 0.93		*p* = 0.10	*p* = 0.48

*p* value at <0.05. Chi-square tests of significance examined intuitive eating by sociodemographic characteristics and weight status.

**Table 4:** Percentage of dieting, weight-control behaviours, and binge eating of respondents (*n* = 367).

Dieting, Weight Control Behaviours, and Binge Eating	Men, % (*n*) *n* = 59	Women, % (*n*) *n* = 308
Currently dieting	9.8 (36)	52.3 (192)
^a^ Healthy weight-control behaviours	16.1 (59)	83.9 (308)
^b^ Unhealthy weight-control behaviours	76.3 (45)	80.7 (246)
Binge eating with loss of control	81.4 (48)	84.7 (261)

Percentage of the respondent. ^a^ Exercise, eating fruits and vegetables, eating less high-fat foods, eating less candy, and drinking sugar-sweetened beverages were all examples of healthy weight-control behaviours. (SSB) and controlling portion sizes. ᵇ Unhealthy weight-management behaviours involve fasting to regulate body weight, eating very little food, using a meal-replacement product (powder or a specific drink), skipping meals, taking diet medications, making oneself vomit, using laxatives, and using diuretics.

**Table 5:** Multiple linear regression analysis on the relationships between intuitive eating and dieting, weight-control behaviours, and binge eating (*n* = 367).

Independent Variable in the Equation	*B*	β	*T*	*p*	R^2^ (F)
Diet	3.299		9.974	0.001	0.34
Binge Eating	0.181	0.57	1.101	0.271	
Weight Control Behaviour	0.151	0.165	3.168	0.002	

Multiple linear regression analysis with a significance level of *p* < 0.05.

## Data Availability

Not applicable.
